# A proposed framework for advancing acute kidney injury risk stratification and diagnosis in children: a report from the 26th Acute Disease Quality Initiative (ADQI) conference

**DOI:** 10.1007/s00467-023-06133-3

**Published:** 2023-09-05

**Authors:** Dana Y. Fuhrman, Natalja L. Stanski, Catherine D. Krawczeski, Jason H. Greenberg, A. Ayse Akcan Arikan, Raj K. Basu, Stuart L. Goldstein, Katja M. Gist, the ADQI 26 workgroup, Rashid Alobaidi, David J. Askenazi, Sean M. Bagshaw, Matthew Barhight, Erin Barreto, Benan Bayrakci, O. N. Ray Bignall, Erica Bjornstad, Patrick Brophy, Jennifer Charlton, Rahul Chanchlani, Andrea L. Conroy, Akash Deep, Prasad Devarajan, Kristin Dolan, Dana Fuhrman, Katja M. Gist, Stephen M. Gorga, Jason H. Greenberg, Denise Hasson, Emma Heydari, Arpana Iyengar, Jennifer Jetton, Catherine Krawczeski, Leslie Meigs, Shina Menon, Catherine Morgan, Jolyn Morgan, Theresa Mottes, Tara Neumayr, Zaccaria Ricci, David T. Selewski, Danielle Soranno, Natalja Stanski, Michelle Starr, Scott M. Sutherland, Jordan Symons, Marcelo Tavares, Molly Vega, Michael Zappitelli, Claudio Ronco, Ravindra L. Mehta, John Kellum, Marlies Ostermann

**Affiliations:** 1 Department of Critical Care Medicine, UPMC Children’s Hospital of Pittsburgh, 4401 Penn Avenue, Suite 2000, Pittsburgh, PA 15224 USA; 2 Department of Pediatrics, Division of Nephrology, UPMC Children’s Hospital of Pittsburgh, Pittsburgh, PA USA; 3 Department of Pediatrics, Division of Critical Care Medicine, Cincinnati Children’s Hospital Medical Center, University of Cincinnati College of Medicine, Cincinnati, OH USA; 4 Department of Pediatrics, Division of Cardiology, Nationwide Children’s Hospital, Ohio State University, Columbus, OH USA; 5 Department of Pediatrics, Division of Nephrology, Yale University Medical Center, New Haven, CT USA; 6 Department of Pediatrics, Division of Critical Care Medicine, Baylor College of Medicine, Texas Children’s Hospital, Houston, TX USA; 7 Department of Pediatrics, Division of Nephrology, Baylor College of Medicine, Texas Children’s Hospital, Houston, TX USA; 8 Department of Pediatrics, Division of Critical Care Medicine, Northwestern University Feinberg School of Medicine, Ann & Robert Lurie Children’s Hospital of Chicago, Chicago, IL USA; 9 Department of Pediatrics, Division of Nephrology & Hypertension, Cincinnati Children’s Hospital Medical Center, Cincinnati, OH USA; 10 Department of Pediatrics, Division of Cardiology, Cincinnati Children’s Hospital Medical Center, University of Cincinnati College of Medicine, Cincinnati, OH USA

**Keywords:** Acute kidney injury, Biomarkers, Pediatrics, Precision medicine, Phenotypes, Diagnosis

## Abstract

Acute kidney injury (AKI) in children is associated with increased morbidity, reduced health-related quality of life, greater resource utilization, and higher mortality. Improvements in the timeliness and precision of AKI diagnosis in children are needed. In this report, we highlight existing, novel, and on-the-horizon diagnostic and risk-stratification tools for pediatric AKI, and outline opportunities for integration into clinical practice. We also summarize pediatric-specific high-risk diagnoses and exposures for AKI, as well as the potential role of real-time risk stratification and clinical decision support to improve outcomes. Lastly, the key characteristics of important pediatric AKI phenotypes will be outlined. Throughout, we identify key knowledge gaps, which represent prioritized areas of focus for future research that will facilitate a comprehensive, timely and personalized approach to pediatric AKI diagnosis and management.

**Supplementary Information:**

The online version contains supplementary material available at 10.1007/s00467-023-06133-3.

## Introduction

The first pediatric-focused Acute Disease Quality Initiative (ADQI) (The Pediatric ADQI; pADQI) meeting was conducted in Napa, CA, USA, as the 26th meeting of the Acute Disease Quality Initiative (ADQI) group (ADQI XXVI) [1]. The current manuscript details the work performed and conclusions drawn by the Risk Assessment and Diagnosis Workgroup, one of the six a priori defined pADQI subgroups.

Acute kidney injury (AKI) is independently associated with increased morbidity and mortality [2]. Its impact may be particularly profound in children [3], who have a longer life expectancy and more time to develop long-term sequelae, including chronic kidney disease (CKD). Despite these serious consequences, AKI management remains largely supportive, and therapeutic strategies are typically instituted only *after* AKI has occurred. In this review, we summarize the existing literature and outline the future of AKI diagnosis in children, including ways to individualize care in real-time.

## Methods

The ADQI process is described in detail elsewhere, including the pADQI parent document [1, 4]. The goal of ADQI is “to provide expert-based statements and interpretation of current knowledge for use by clinicians according to professional judgment and identify evidence care gaps to establish research priorities” [5]. In the 26th ADQI, we addressed the primary question of “What are the unique considerations for AKI risk stratification and diagnosis in children?” This question served as the foundation for the following consensus statements.

## 26th ADQI consensus statement (Recommendation)

*Validated tools which incorporate both patient characteristics and exposures and also interface with the local health care environment should be utilized to estimate AKI risk in children, including assessment of objective measures of kidney fitness in at-risk children prior to a predictable or planned intervention* [1]*.*

### High-risk diagnoses and exposures

**Fig. 1:**
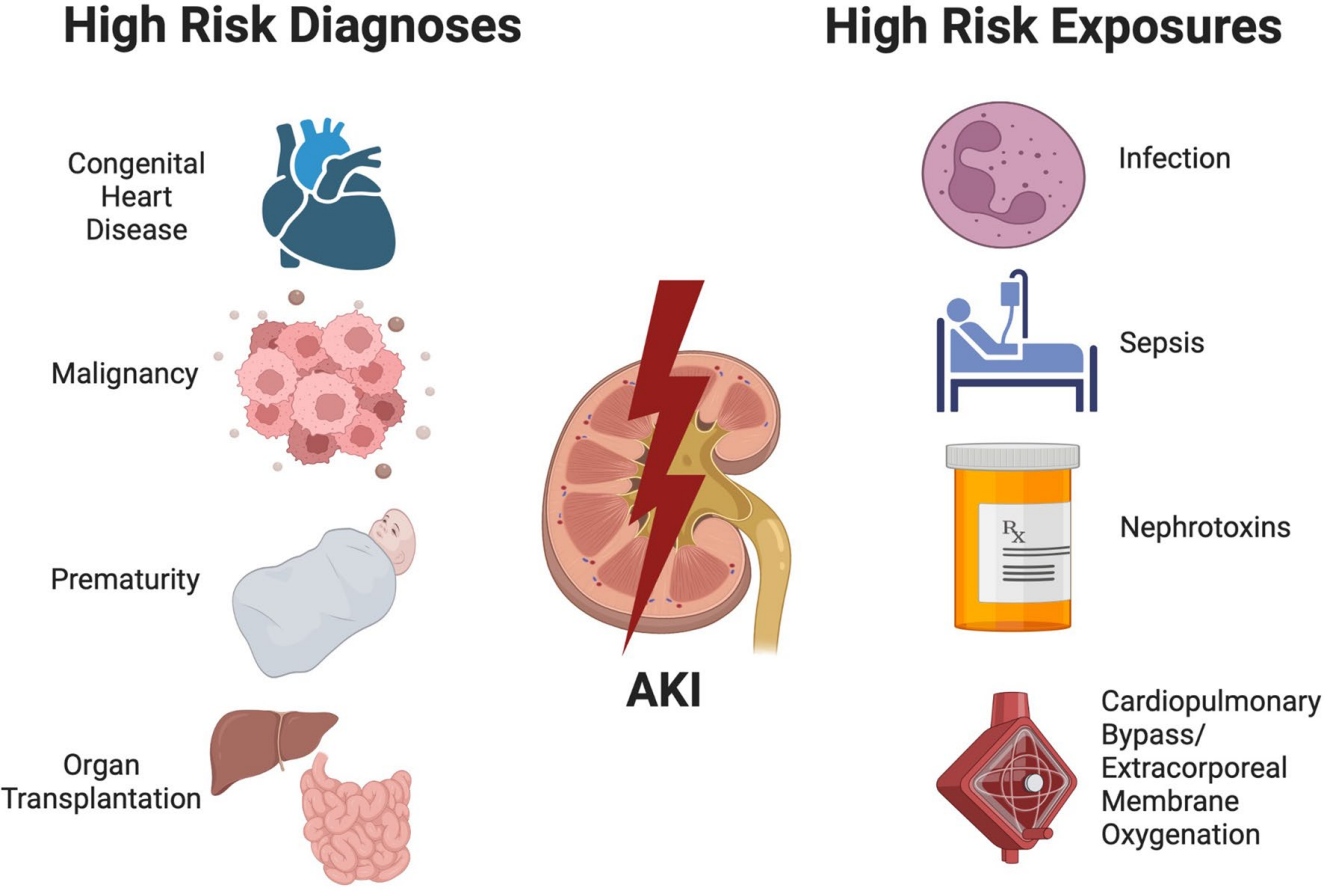
Diagnoses and exposures that are associated with a greater risk for acute kidney injury. There are distinct patient diagnoses and exposures that increase the risk for acute kidney injury in hospitalized children

Accurate identification of children at risk for AKI is the critical first step to improving outcomes. Children in any intensive care unit (ICU) have higher rates of AKI compared to non-ICU patients [6] with reported incidences of 27% in the pediatric ICU [3], 30% in the neonatal ICU [6], and 54% in the cardiac ICU [7]. In these populations, the risk for AKI is highest in critically ill children with sepsis, congenital heart disease, malignancies, and those receiving invasive mechanical ventilation [8–10]. Importantly, very low birth weight infants are especially vulnerable, with AKI rates up to 48% [11–13]. It is crucial that those clinicians caring for patients in neonatal, general pediatric, and cardiac ICUs maintain heightened surveillance for AKI. These risk factors are summarized in Fig. 1.

Maintaining awareness of exposures that increase the risk for AKI in hospitalized children is key to AKI prevention (Fig. 1). A common inciting risk factor for AKI in children is nephrotoxic medication exposure [14]. In particular, the combination use of certain agents such as vancomycin and piperacillin-tazobactam may be associated with an increased risk for AKI [15]. In non-critically ill children, the Nephrotoxic Injury Negated by Just-in-time Action (NINJA) collaborative developed consensus for the classification of nephrotoxic medications [16]. Recognition of high nephrotoxic medication burden as an AKI risk factor along with enhanced monitoring in the inpatient setting can decrease the rates of nephrotoxic medication-associated AKI [14, 17, 18].

### Assessment of kidney fitness: subclinical kidney injury and kidney functional reserve

AKI diagnosis currently relies on changes in functional kidney biomarkers serum creatinine (SCr) and/or urine output, which are often delayed and imprecise markers of kidney function, particularly in patients with a rapidly changing glomerular filtration rate (GFR). A two-decade research focus has been directed at identifying novel tubular injury biomarkers to diagnose AKI sooner and more precisely, particularly after a high-risk exposure. Yet a child’s AKI risk would be assessed ideally *prior* to high-risk exposure. Since more than 70% of children admitted to a pediatric ICU in the USA have chronic healthcare needs [19], opportunities exist prior to hospitalization to evaluate a child’s kidney fitness. Kidney fitness refers to an adaptive ability to respond well to kidney stress and, therefore, show a decreased risk for both AKI and a decline in GFR over time [20]. Proactive assessment of kidney fitness includes standard measures of kidney function and injury obtained prior to planned events in patients with a high-risk diagnosis and/or exposure.

Kidney fitness assessment will likely move beyond traditional markers of kidney function. Quantification of kidney functional reserve has received renewed interest, with emerging evidence that adults with reduced kidney functional reserve prior to cardiac surgery are more likely to develop post-operative AKI and be at increased risk for CKD [21, 22]. This concept warrants further study in children. Pre-operative values of candidate biomarkers such as urinary DKK3 [23], uromodulin [24], and serum FGF-23 [25] show promise for predicting AKI after cardiac surgery as possible markers of kidney fitness.

### Validated tools for assessing AKI risk in hospitalized children

Derived and validated specifically for critically ill pediatric patients, the Renal Angina Index (RAI) incorporates demographic characteristics and real-time patient data to predict severe AKI 72 h after ICU admission (Supplementary Fig. 1) [26], with a recent meta-analysis of over 3000 patients demonstrating a pooled AUROC of 0.88 (95% CI 0.85–0.91) [27]. Importantly, this simple and pragmatic tool has been assessed in resource-limited settings with good predictive performance [28]. Furthermore, targeted measurement of urinary biomarkers like neutrophil gelatinase-associated lipocalin (NGAL) improves risk stratification afforded by the RAI [29, 30]. The RAI has now also been modified and operationalized for early prediction of AKI in children in the emergency room [31], with sepsis [32], and after cardiac surgery [33]. Similar modifications to the RAI are needed for the neonatal ICU population, as well as for oncologic and post-transplant patients outside of the ICU setting.

However, a key limitation of the RAI is that it is obtained at a single, cross-sectional point in time from ICU or emergency department admission. It does not take into consideration changes in fluid status, SCr, and nephrotoxin exposure that are likely to occur over the course of a patient’s hospital stay. The Fluid Overload Kidney Injury Score (FOKIS) is a more recently developed score that is continuously calculated with the addition of any new data elements to the electronic health record (EHR) [34]. This four-dimensional score includes a standardized pediatric AKI assessment of changes in urine output and SCr, nephrotoxic medication exposure, and assessment of fluid overload. The score has been studied prospectively and shown to be associated with mortality and length of stay in a general pediatric ICU cohort [34] but requires validation in other patient groups.

### A framework for comprehensive AKI risk assessment in children

**Fig. 2:**
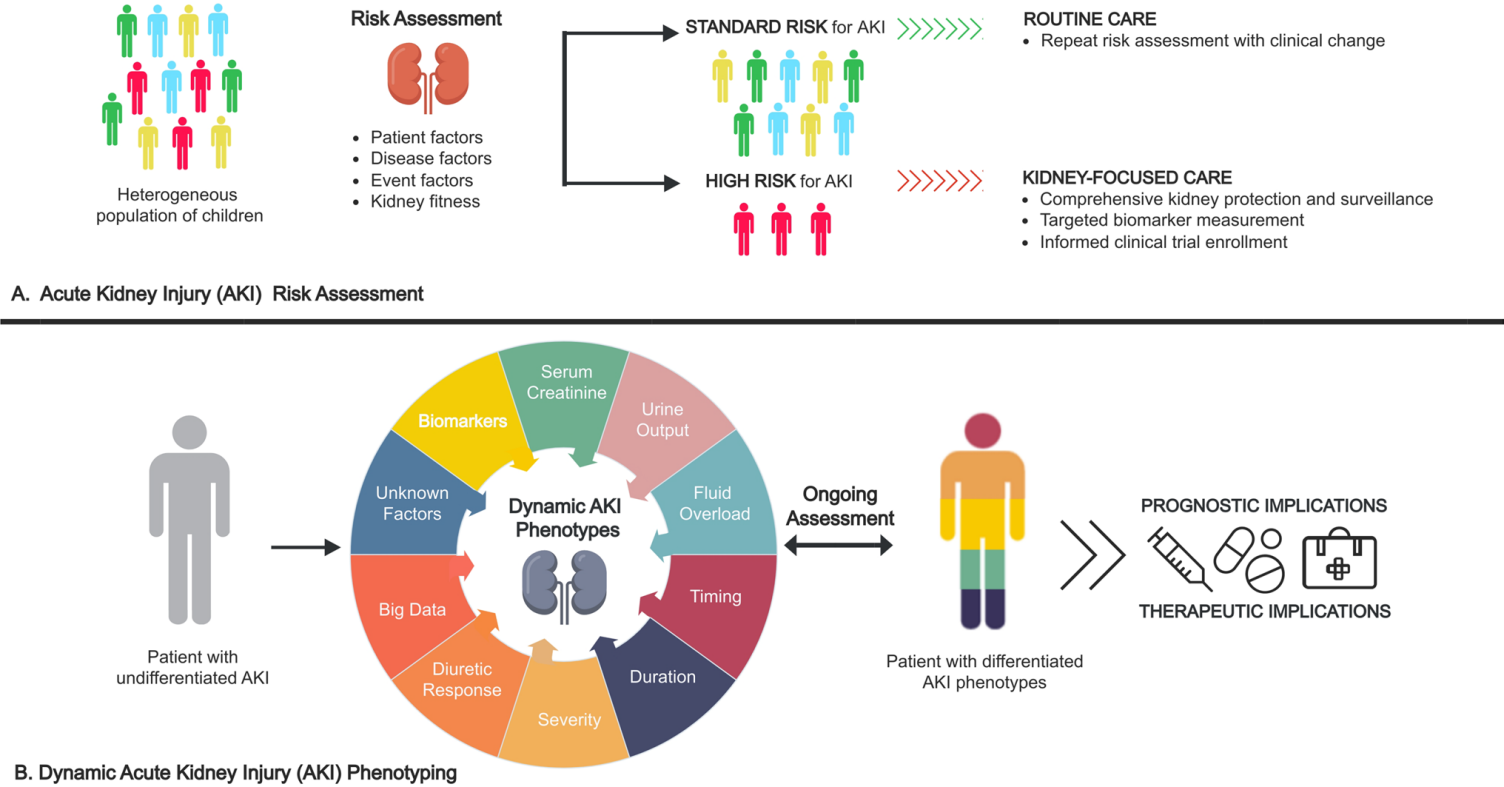
Acute kidney injury risk assessment and dynamic phenotyping. **A** Acute kidney injury risk assessment. A comprehensive acute kidney injury risk assessment should occur in children admitted to the hospital, prior to planned high-risk exposures, and repeated with clinical changes. This assessment allows for individual risk stratification that can focus resources and identify patients appropriate for more intensive, kidney-focused monitoring and care. **B** Dynamic acute kidney injury phenotyping. Combined with individual susceptibility, multiple elements contribute to discernible acute kidney injury phenotypes in affected children which may have prognostic and therapeutic implications. This phenotype is dynamic and may change over the course of illness, highlighting the importance of ongoing assessment. Source: Acute Disease Quality Initiative

At the time of hospital admission (or before, if planned), providers should complete a history and chart review that includes an evaluation for exposures and susceptibilities for AKI (Fig. 2A). Patients deemed to be at standard risk for AKI would undergo repeat AKI risk assessment with clinical changes during their hospital course, as their risk profile changes. Patients identified as high risk based on this assessment would undergo kidney-focused care including, but not limited to, more frequent SCr monitoring, careful attention to volume status (including close urine output monitoring, which may require foley catheter placement in some instances), and discontinuing or replacing nephrotoxic medications as soon as it is medically appropriate. Importantly, there is a crucial need for utilizing prognostic enrichment in the employment of AKI prevention and treatment strategies. However, interventions aimed at prevention of AKI in pre-identified high-risk populations have had variable success. A recent meta-analysis of 13 studies found that implementation of AKI care “bundles” in hospitalized patients reduces moderate–severe AKI, mainly driven by studies in an ICU setting [35]. In addition, there are reports of successful AKI care bundles in adults after cardiopulmonary bypass surgery [36] and non-cardiac surgery [37]. Bundle components were heterogeneous, and compliance to bundles were variable, limiting the ability to draw conclusions. There is a need for investigations exploring the impact of feasible AKI prevention bundles in pediatric patients that take into consideration the unique aspects of this patient population and their care.

Data on risks for pediatric AKI in the community or outpatient setting are scarce [6, 38]. Notably, in limited resource settings, AKI is typically community-acquired rather than hospital-acquired [39]. In contrast to focusing on AKI care bundles enacted at the time of hospital admission, efforts aimed at providing clean drinking water, sanitation, and access to healthcare are crucial in preventing AKI in resource-limited countries [39].

### Making the timely diagnosis of AKI in children: expanding our diagnostic tools

The last two decades have witnessed a concerted effort to identify novel biomarkers to detect and predict AKI earlier than functional markers (i.e., SCr and urine output) alone, within a therapeutic “window of opportunity.” When available, biomarkers of kidney damage or stress may allow further insight into AKI risk and phenotype (Table 1). Ideally, therapies to prevent progression or severity of AKI would be employed before both structural and functional biomarkers are elevated. Unfortunately, despite good to excellent performance in multiple studies [40–42], most structural biomarkers are not routinely used clinically due to lack of biomarker assay availability or regulatory approval, or because of cost. Highlighting further the need to expand upon existing AKI diagnostic tools, in many low- and middle-income countries, even SCr testing is unavailable [43]. In these settings, point-of-care urine NGAL and salivary urea nitrogen testing have been proposed as feasible alternatives for the diagnosis of AKI in children [44].

**Table 1:** Biomarkers of acute kidney damage and stress

Biomarker	Proposed role in the kidney	Clinical settings where the biomarker has been evaluated for predicting AKI in pediatrics	Number of pediatric studies cited in PubMed in the last 10 years*
NGAL	Induced and secreted by distal tubular epithelial cells after injury	Burn patients, critically ill children, critically ill neonates, cystic fibrosis, emergency room, hemolytic uremic syndrome, kidney transplant, non-critically ill hospitalized children, post cardiac surgery, post radiographic contrast, stem cell transplantation, urinary tract infection	183
KIM-1	Up-regulated in proximal tubular epithelial cells after injury	Burn patients, critically ill children, critically ill neonates, cystic fibrosis, emergency room, kidney transplant, non-critically ill hospitalized children, post cardiac surgery, post radiographic contrast, stem cell transplantation, urinary tract infection	75
[TIMP -2]•[IGFBP7]	Expressed by tubular epithelial cells in response to early cellular injury	Critically ill children, critically ill neonates, post cardiac surgery	14

Table adapted from: Fuhrman DY. The Use of Diagnostic Tools for Pediatric AKI: Applying the Current Evidence to the Bedside. *Pediatr Nephrol.* 2021;36(11):3529–3537

Abbreviations: *AKI* acute kidney injury, *NGAL* neutrophil gelatinase-associated lipocalin, *KIM* kidney injury molecule, *TIMP* tissue inhibitor of metalloproteinases, *IGFBP* insulin-like growth factor-binding protein

^*^Includes exclusively clinical studies, meta-analyses, and systematic reviews exploring the use of the biomarker to predict kidney outcomes

The widespread implementation of EHRs in resource-rich settings provides an opportunity to leverage electronically accessible data to enhance risk stratification and AKI detection at the bedside to improve outcomes. Automated alerts and associated real-time clinical decision support (CDS) within the EHR can facilitate the identification of at-risk patients and notify clinicians of abnormal measurements that may predict AKI development or progression [43]. The NINJA study demonstrates that standardized AKI risk assessment and surveillance in non-critically ill hospitalized children receiving nephrotoxic medications can lead to sustained reductions in both AKI incidence and severity [45]. As part of the NINJA initiative, a CDS program was implemented to identify those at risk for AKI, directing daily SCr surveillance and medication dose adjustment or discontinuation. The initial single-center evaluation of this program demonstrated a 38% reduction in nephrotoxic medication exposure and 64% reduction in AKI [45], and it has subsequently been disseminated and implemented at more than a dozen children’s hospitals across the USA [14]. While these data are promising, it is important to note that the epidemiology of AKI and the resources available may be different in developing countries, and thus specific AKI CDS strategies should be developed and focused accordingly. Additionally, given the heterogeneity of the pediatric population, alerts should be customizable to reflect age-adjusted reference ranges and patient-specific parameters [46, 47].

While AKI alert systems may improve the timeliness of AKI diagnosis, they likely provide the greatest benefit if they also suggest targeted interventions to limit its progression and negative sequelae. For example, in a study of over 3000 hospitalized adults, AKI alerts with recommendations for a targeted nephrology consult led to a 25% decrease in severe AKI rates [48]. Similarly, a pediatric study demonstrated implementation of an AKI alert with CDS to guide patient evaluation and management resulted in a decrease in AKI stage and a higher proportion of nephrotoxic medication adjustment and changes in fluid management compared to the pre-CDS phase [49]. Though these studies highlight the potential of EHR-based AKI alerts and CDS, it is important to note that these data are balanced by a recent large, multi-center, randomized controlled trial demonstrating that an EHR AKI alert and associated CDS failed to decrease AKI progression, and even resulted in higher risk of worsening AKI or death at some centers [46]. Clearly, these conflicting study results highlight the importance of conducting further prospective clinical trials examining the role of EHR-based AKI alerts and CDS in children, ensuring the incorporation of considerations unique to the pediatric population.

## 26th ADQI consensus statement (Suggestion)

*Unique AKI phenotypes in children may overlap and change over time. Differentiating AKI phenotype(s) informs prognosis and has the potential to guide therapeutics* [1].

### A precision medicine primer: phenotypes, endotypes, and enrichment

AKI is a heterogeneous syndrome with multiple etiologies, clinical manifestations, and biological underpinnings. Therefore, efforts are underway to identify and characterize unique subsets of AKI, in order to advance care by applying the tenets of precision medicine, as has been successfully done in other heterogeneous pediatric syndromes like sepsis and acute respiratory distress syndrome (ARDS) [50, 51]. Broadly, precision medicine refers to preventive, diagnostic and/or treatment strategies that take individual patient and/or disease factors into account [52, 53]. Key to such an approach is the identification of unique disease *phenotypes*, which are defined by clinically observable characteristics of the disorder of interest [54]. However, as our ability to incorporate molecular and biochemical information into these unique subsets has improved, the term *endotype* describes a unique phenotype defined by a distinct underlying pathobiology more appropriately [54]. Thus, the identification of *endotypes* represents a key step in phenotyping work, as it ties clinically defined clusters of patients to underlying biology and offers a mechanism for identifying appropriate therapies tailored to the individual patient.

This concept of identifying the right therapy for the right patient is an example of *enrichment*, a tenet of precision medicine, and the goal of heterogeneous disorder phenotyping [52, 53]. Enrichment strategies can be *prognostic* (i.e., selecting patients with a higher likelihood of having a disease-related outcome of interest, such as development of AKI or need for kidney replacement therapy (KRT)), or *predictive* (i.e., selecting patients more likely to respond to a therapy on the basis of biology) [52]. Thus, when defining clinically important AKI phenotypes in children, it is important to consider how a phenotyping strategy facilitates either prognostic or predictive enrichment. We propose AKI phenotyping in children focuses on the following: (1) identifying children who are more likely to suffer *meaningful* outcomes of interest (i.e., prognostic enrichment), and (2) identifying children with shared underlying biology who may benefit from a specific therapy (i.e., predictive enrichment). Supplementary Fig. 2 outlines these proposed priorities for pediatric AKI phenotyping.

### Pediatric AKI phenotypes: current state of the art

Much of the AKI phenotyping work to date facilitates prognostic enrichment. In particular, several different strategies have been utilized to identify patients at high risk for severe AKI (i.e., ≥ Kidney Disease Improving Global Outcomes (KDIGO) Stage 2), persistent AKI (present for ≥ 48 h), and those more likely to require KRT [55–63]. Validation of AKI phenotypes to reliably identify at-risk patients will direct patient and family counseling, targeted implementation of intensive kidney supportive care, and clinical trial enrichment for future studies examining novel AKI therapies. The latter is of particular importance in children given the relatively small patient population compared to adults.

#### Urine output phenotypes

Urine output is one of two functional biomarkers in the KDIGO criteria for AKI diagnosis. Until recently, little data existed regarding its impact on AKI epidemiology and outcomes in children [64]. A post hoc analysis of the multi-center, multinational Assessment of Worldwide Acute Kidney Injury, Renal Angina and Epidemiology (AWARE) study demonstrated nearly 20% of critically ill children with AKI were diagnosed by urine output criteria only, and that these patients had similar negative outcomes to those diagnosed only by SCr criteria [60]. Furthermore, compared to patients without AKI, meeting criteria for AKI by *both* SCr and urine output increased the relative risk for receiving KRT to 165.7 (95% CI, 86.3–318.2) (*p* < 0.01) from 9.1 (95% CI, 3.9–21.2) in those diagnosed by SCr alone [60]. A separate post hoc analysis of AWARE developed a summative AKI score that added AKI stage by SCr to AKI stage by urine output, showing increasing AKI score was associated with worse outcomes, including KRT use, prolonged length of stay, AKI non-recovery and mortality [61].

#### Fluid accumulation phenotypes

Excessive positive fluid balance or fluid overload (FO) has been well demonstrated to be associated with increased morbidity and mortality in critically ill children [65]. One study of 149 critically ill children combined FO with functional AKI staging by SCr or urine output to delineate unique AKI phenotypes (AKI-/FO-, AKI + /FO-, AKI-/FO + , AKI + /FO +). The 24% of patients who developed ≥ 20% fluid accumulation by ICU Day 3 experienced worse outcomes (including prolonged length of stay and mortality), irrespective of functional AKI status [58]. The concepts surrounding fluid accumulation and associated outcomes are discussed more in depth by pADQI Work Group 3.

#### Severity and duration phenotypes

The ADQI 16 outlined definitions of transient AKI (sustained reversal within 48 h of onset) versus persistent AKI (≥ 48 h in duration) in adults, recognizing that outcomes in the latter subset of patients were worse [66]. An assessment of AKI duration-based phenotypes in a cohort of children with sepsis from AWARE demonstrated patients with persistent AKI had fewer ICU-free days and more complex ICU course than patients with transient AKI [55]. Additionally, combination of AKI duration severity delineated four unique AKI phenotypes (mild–transient, mild–persistent, severe–transient, severe–persistent) that had differential outcomes [56]. Two other studies showed severity and duration phenotypes in children following cardiac surgery to be associated with worse outcomes among those with persistent AKI [59, 67].

#### Response to loop diuretics

A standardized assessment of diuretic responsiveness, termed the furosemide stress test (FST) predicts the progression of AKI in critically ill patients [68]. Retrospective pediatric studies have examined the association between furosemide responsiveness and AKI-related outcomes following cardiac surgery [62, 63]. Urine output response to furosemide in the first 24 h following cardiopulmonary bypass predicted cardiac surgery-associated AKI, peak FO, and other poor outcomes, including mortality [63]. Lower urine flow rates in children following cardiac surgery at 2 and 6 h after furosemide were associated with AKI and prolonged length of stay [62]. While data from these studies has been extrapolated to propose 3 ml/kg/h urine flow rate to predict AKI progression in children [69], larger, prospective studies are still needed to validate this threshold, and to fully understand the role of loop diuretic responsiveness in identifying a unique AKI phenotype in children.

#### Biomarker-based phenotypes

The ADQI 10 proposed a new AKI model to integrate functional markers and damage biomarkers to delineate unique biomarker-based AKI phenotypes [70]. In children after cardiopulmonary bypass, investigators showed that combining a functional biomarker like cystatin C with a tubular damage biomarker like NGAL was superior to changes in SCr for predicting the duration of AKI [56]. A single-center study derived SCr and NGAL-based AKI phenotypes (NGAL-/SCr-, NGAL + /SCr-, NGAL-/SCr + , NGAL + /SCr +) on the day of pediatric ICU admission and examined their associations with AKI outcomes at Day 3 [6]. Patients with functional AKI without evidence of tubular damage (NGAL-/SCr +) were more likely to have transient AKI (i.e., resolved by Day 3), while those with evidence of tubular damage (i.e. NGAL +) were more likely to have AKI on Day 3, regardless of functional biomarker status (i.e., SCr + or SCr-) [57]. Similar to work in adults, this study highlighted the significance of subclinical AKI (i.e. tubular injury biomarker positive without functional biomarker elevation, NGAL + /SCr-), as these patients had worse outcomes compared to biomarker negative patients [57]. Since AKI biomarkers such as NGAL remain clinically unavailable at many pediatric centers, more work is needed to validate these findings and to elucidate when and in whom to obtain tubular damage biomarkers.

### Pediatric AKI phenotypes: the gaps

While the above-described frameworks outline static, one, or two-factor-based AKI phenotypes, it is likely that more than one may be present in a child with AKI at any given time, and that this unique combination of phenotypes may change over the course of illness (Fig. 2B). Thus, key knowledge gaps in our understanding of AKI phenotypes relate to delineating the synergistic and time-dependent effects of these phenotypes on patient outcomes, an important area of future research.

Finally, a second major gap in pediatric AKI phenotyping to date remains strategies that facilitate predictive enrichment (Supplementary Fig. 2). While we may be able to use some of the constructs outlined above to make therapeutic decisions (for example, a patient with NGAL elevation, who has FO and is not responsive to loop diuretics, may be an appropriate candidate for consideration of early KRT), less is known about the biological underpinnings of AKI phenotypes, which limits our ability to provide individualized, biology-informed treatments. Lessons can be learned from previous work in pediatric sepsis and ARDS, where both unique endotypes and phenotypes have been delineated that may identify those most likely to respond to a given therapy (i.e., stress corticosteroids in septic shock) [50, 51]. Ultimately, a phenotyping approach that combines prognostic enrichment (i.e., AKI risk stratification) and predictive enrichment (i.e., identification of a phenotype or endotype likely to respond to a specific therapy) will be needed to inform enrollment in future clinical trials aimed at identifying successful AKI therapies.

## Conclusions

Given that AKI is associated with a higher risk of morbidity, mortality, and health care resource utilization, establishing a timely and accurate diagnosis is essential. It is necessary that those caring for children admitted to the hospital have a knowledge of high-risk diagnoses and exposures that are associated with pediatric AKI, and that community-based providers are given tools to identify and assess at-risk patients prior to hospitalization. It has become evident that we cannot solely rely on traditional markers of kidney function, such as urine output and SCr, for diagnosing AKI. The use of novel biomarkers incorporated with a validated AKI risk assessment tool like the RAI, can improve our ability to predict AKI. Additionally, tubular damage biomarkers along with factors such as fluid status, diuretic response, AKI severity, and duration may help us to identify unique pediatric patient AKI phenotypes. Ultimately, knowledge of these dynamic phenotypes show tremendous promise to guide clinicians in making prognostic and therapeutic decisions for children with AKI.

### Supplementary Information

Below is the link to the electronic supplementary material.

Supplementary Figure 1. The Renal Angina Index. Derived and validated in critically ill children, the renal angina index is calculated 12 hours after intensive care unit admission from demographic and clinical data. A score of 8 or higher defines “renal angina” and has been demonstrated to predict severe acute kidney injury 72 hours later. (JPG 360 KB)

Supplementary Figure 2. Priorities for pediatric acute kidney injury phenotyping. Acute kidney injury phenotyping efforts in children should focus on identifying those most likely to suffer meaningful outcomes of interest (i.e. prognostic enrichment) and identifying those with shared underlying biology that may direct therapy (i.e. predictive enrichment). Identifying these unique subsets of patients has the ability to inform care and improve outcomes, including via targeted clinical trial enrollment and the delivery of patient-specific biology-based therapies. (JP2 88 KB)
