# Supplementary figures and images for: A proposed framework for advancing acute kidney injury risk stratification and diagnosis in children: a report from the 26th Acute Disease Quality Initiative (ADQI) conference
Source: Pediatr Nephrol. 2023 Sep 5;39(3):929–39. doi: 10.1007/s00467-023-06133-3 (PMC10817991; doi:10.1007/s00467-023-06133-3)

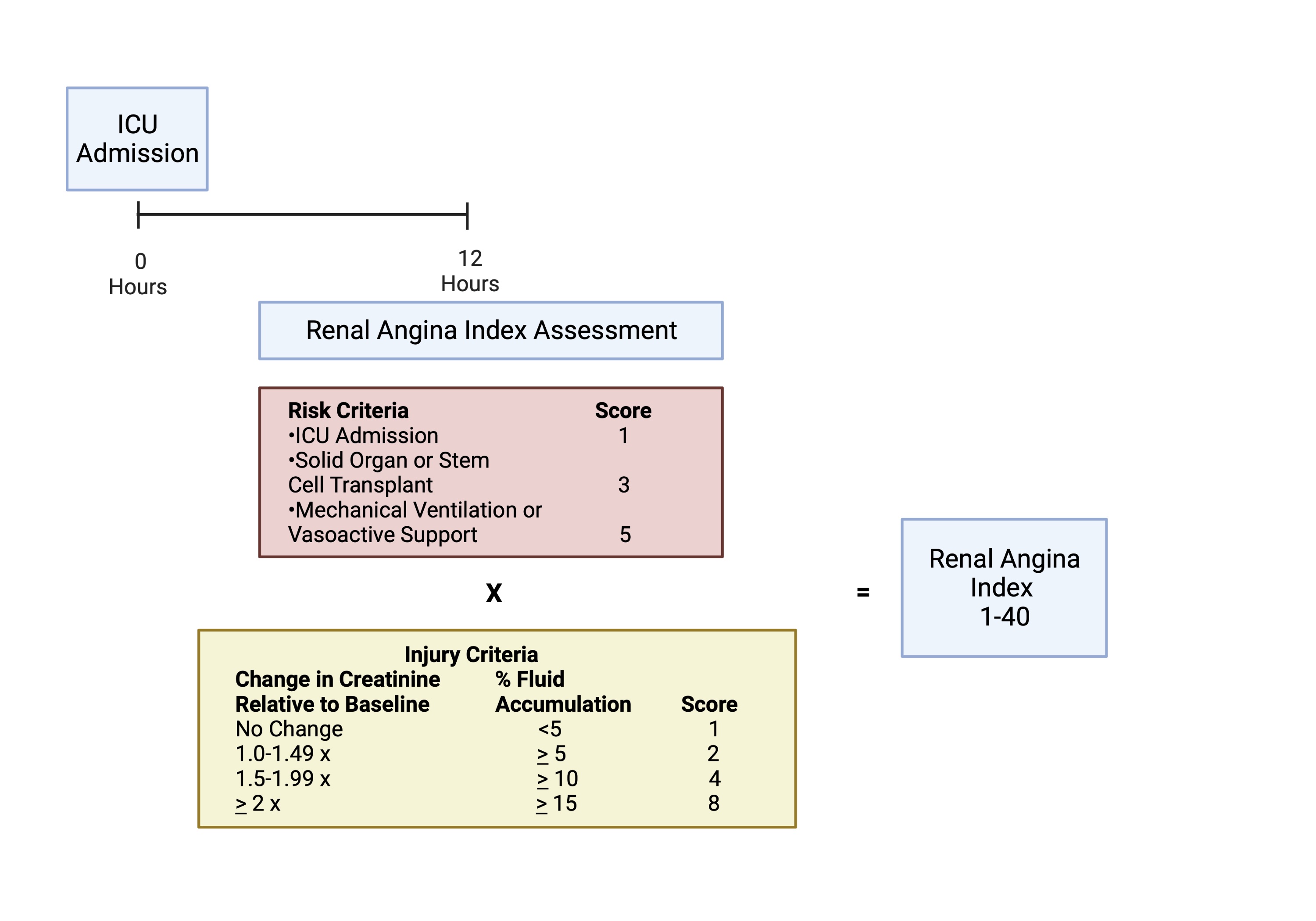

Supplement: Supplementary file 1 — Supplementary Figure 1. The Renal Angina Index. Derived and validated in critically ill children, the renal angina index is calculated 12 hours after intensive care unit admission from demographic and clinical data. A score of 8 or higher defines “renal angina” and has been demonstrated to predict severe acute kidney injury 72 hours later. (JPG 360 KB) [file 467_2023_6133_MOESM1_ESM.jpg]
